# Curcumin prevents neuronal loss and structural changes in the superior cervical (sympathetic) ganglion induced by chronic sleep deprivation, in the rat model

**DOI:** 10.1186/s40659-020-00300-8

**Published:** 2020-07-10

**Authors:** Mahboobeh Erfanizadeh, Ali Noorafshan, Mohammad Reza Namavar, Saied Karbalay-Doust, Tahereh Talaei-Khozani

**Affiliations:** 1 Department of Anatomical Sciences, School of Medicine, Shiraz University of Medical Sciences, Shiraz, Iran; 2 Histomorphometry and Stereology Research Centre, Shiraz University of Medical Sciences, 71348-45794 Shiraz, Iran; 3 Clinical Neurology Research Center, Shiraz University of Medical Sciences, 71348-45794 Shiraz, Iran; 4 Laboratory for Stem Cell Research, Department of Anatomical Sciences, Shiraz University of Medical Sciences, Shiraz, Iran

**Keywords:** Chronic sleep deprivation, Superior cervical ganglion, Curcumin, Stereology, Apoptosis, Spatial arrangement of neurons

## Abstract

**Background:**

In modern societies, sleep deprivation is a serious health problem. This problem could be induced by a variety of reasons, including lifestyle habits or neurological disorders. Chronic sleep deprivation (CSD) could have complex biological consequences, such as changes in neural autonomic control, increased oxidative stress, and inflammatory responses. The superior cervical ganglion (SCG) is an important sympathetic component of the autonomic nervous system. CSD can lead to a wide range of neurological consequences in SCG, which mainly supply innervations to circadian system and other structures. As the active component of Curcuma longa, curcumin possesses many therapeutic properties; including neuroprotective. This study aimed to evaluate the effect of CSD on the SCG histomorphometrical changes and the protective effect of curcumin in preventing these changes.

**Methods:**

Thirty-six male rats were randomly assigned to the control, curcumin, CSD, CSD + curcumin, grid floor control, and grid floor + curcumin groups. The CSD was induced by a modified multiple platform apparatus for 21 days and animals were sacrificed at the end of CSD or treatment, and their SCGs removed for stereological and TUNEL evaluations and also spatial arrangement of neurons in this structure.

**Results:**

Concerning stereological findings, CSD significantly reduced the volume of SCG and its total number of neurons and satellite glial cells in comparison with the control animals (*P *< 0.05). Treatment of CSD with curcumin prevented these decreases. Furthermore, TUNEL evaluation showed significant apoptosis in the SCG cells in the CSD group, and treatment with curcumin significantly decreased this apoptosis (*P *< 0.01). This decrease in apoptosis was observed in all control groups that received curcumin. CSD also changed the spatial arrangement of ganglionic neurons into a random pattern, whereas treatment with curcumin preserved its regular pattern.

**Conclusions:**

CSD could potentially induce neuronal loss and structural changes including random spatial distribution in the SCG neurons. Deleterious effects of sleep deprivation could be prevented by the oral administration of curcumin. Furthermore, the consumption of curcumin in a healthy person might lead to a reduction of cell death.

## Background

Mammals spend about one-third of their life span asleep. Sleep is a salient feature of health and wellbeing that impacts multiple aspects of development, tissue regeneration, and learning [1]. One of the most common chronic stresses that have become a concern in modern society is sleep deprivation. Sleep deprivation is described as a state of inadequate quantity or quality of sleep [2]. Chronic sleep deprivation (CSD) is able to induce diverse biological effects, such as increased oxidative stress, inflammatory responses, memory impairment [3], and alteration in the autonomic nervous system [4]. Additionally, autonomic nervous dysfunction has also been found to be a risk factor for atherosclerosis and cardiovascular disease [5]. Moreover, investigations have shown that partial sleep deprivation activates DNA damage response [6] and neuronal death, suppresses neuronal proliferation [7], reduces hippocampal gliogenesis [8], and dendritic spines and length in different parts of the brain [9, 10].

The superior cervical ganglion (SCG) is the most rostral ganglion in the sympathetic chain and located deep to the carotid sheath, which plays a major role in coordinating the circadian cycle by innervation of pineal gland via the Nervi conarii [11, 12]. This ganglion provides sympathetic innervation to diverse areas including anterior hypophysis, the hypothalamus [13, 14], choroid plexus, carotid body [15, 16], eye, the lacrimal, salivary, thyroid glands, and cephalic blood vessels, and pilomotor muscles of the cheek and the skin of the forehead that lie rostral to the ganglion [12, 17]. In addition, its postganglionic fibers join the cardiac plexus as the cardiac nerve, or join the 9th, 10th, and 11th cranial nerves and also form the grey rami communicants for spinal nerves C1–C4 [18].

Previous evidence has shown that the superior cervical ganglion can control vasoconstriction of blood vessels in the brain [19], alter cerebrospinal fluid production [20], and change melatonin secretion and thereby affects its rhythm [21].

Because, the SCG provides innervation to many structures and organs in the neck and head, changes in the number of its neuronal or glial cells, or even cell death due to chronic sleep deprivation, might affect the function of various organs that receive their sympathetic innervations from this structure.

Previous studies have shown that sleep deprivation can lead to structural changes and apoptosis and loss of neurons in different parts of the brain such as the prefrontal cortex, hippocampus [9], and respiratory nuclei of the brain stem [22]. A recent report on humans showed that gray matter volume is reduced upon sleep disturbance [23]. Neuronal death in the dorsal raphe nucleus [24] and locus coeruleus has been also observed due to REM sleep loss [25].

Hitherto, the impact of sleep deprivation on histology of SCG and its possible changes has not been studied. Due to structural changes in many parts of the central nervous system as a result of sleep deprivation, we hypothesized that CSD might have detrimental effects on the SCG. As mentioned above, damage to this ganglion may adversely have effects on the function of many organs receiving sympathetic innervation from this structure.

Since, CSD impairs the nervous system structure and possibly SCG, we have used a Food and Drug Administration (FDA)-approved agent, curcumin, to prevent CSD possible adverse effects. Curcumin is the most dynamic component of rhizomes of Curcuma longa of the ginger family. Amongst the numerous natural medications, curcumin has gained considerable attention due to its profound remedial values [26], extensively due to its bio-functional properties, especially antioxidant, anti-inflammatory, anti-aging, neuroprotective [27], neurogenesis and memory-enhancing activities [28]. Another benefit of this herbal extract is that it can prevent neuronal death, which was demonstrated by Jia et al. [29]. Previous studies showed that curcumin can recover the volume, neuronal number, and reconstruction in the hippocampus in the REM-sleep deprivation [30]. Our goal in this study was to find whether curcumin has similar neuroprotective effects on the SCG, such as the effects on the brain and other parts of the nervous system. Hence, we assessed the impact of chronic sleep deprivation on the volume, neuronal and glial numbers, and cell death in the superior cervical ganglion, and the possible protective effects of curcumin on these changes. For this purpose, unbiased stereological methods and TUNEL assay were applied to provide the above quantitative insights.

In addition to structural, cell number, or other stereological parameters changes that have important roles in the function of an organ, the spatial distribution of cells in that organ has been recently considered as an important factor. For example, in some experimental conditions like cerebral [31] and myocardial [32] ischemia, tissue cells lost their normal spatial distributions. The tessellation provides information concerning spatial distribution; the areas of the Voronoi polygons do not vary much when the cells are regularly distributed. On the contrary, small and large polygons are found when cellular clusters are present. As mentioned, the death of neurons or glial cells, irregularities in dendritic arborization and change in dendritic length probably due to sleep deprivation, might change the distance between cells and the area of ​​the Voronoi polygons after CSD, in turn, may have effects on SCG and thereby its related structures function. Therefore not only the number of ganglionic neurons but also the spatial arrangement of these neurons is important in the balance function of SCG. In the previous studies on the CSD, most of the histomorphological evaluations have been focused on the number of neurons [18], and less attention has been given to the spatial distribution of neurons. Therefore, in addition to evaluation of the number of neuron and glia and cell death as well as the volume of the SCG, for the first time, the spatial arrangement of neurons in this structure and their possible changes following sleep deprivation as well as curcumin treatment on these changes will be assessed.

## Materials and methods

### Animals and drugs

A total of 36 male Sprague–Dawley adult rats (250–300 g) were purchased from the Comparative and Experimental Medical Center at Shiraz University of Medical Sciences (SUMS, Iran). All procedures were performed based on the guidelines on the standard for animal care, which was approved by the local Ethical Committee of SUMS (IR.SUMS.REC.1396.S630). Animals were kept under standard conditions, temperature (22 ℃–24 °C), and a 12:12 h light–dark cycles, with ad libitum access to food and water during the 21 days of the experiment. They were randomly assigned into six groups (n = 6/group) including control, curcumin, sleep-deprived and sleep-deprived + curcumin, grid floor, and grid floor + curcumin groups. The control and curcumin rats were placed in cages with a hard floor and sawdust was used as bedding. Sleep-deprived and sleep-deprived + curcumin rats were placed in a water tank with multiple platforms to induce CSD. As a suitable control group for the CSD, the grid floor and grid floor + curcumin animals were kept in a water tank with a stainless steel grid mesh put on the round platforms [33].

All animals in curcumin-treated groups received one ml curcumin **(**Sigma-Aldrich, Germany) at the dose of 100 mg/kg/day dissolved in phosphate-buffered saline (PBS), via oral gavage for 21 days. Other groups were fed the same volume of PBS.

### Induction of chronic sleep deprivation

The paradoxical sleep-deprivation was induced using the modified multiple platform method (MMPM) for 21 days. For this purpose, each group of rats was placed in a Plexiglas water tank (125 × 45 × 45 cm) containing 14 round small platforms (6.5 cm in diameter). The method of limiting sleep in this study was rapid eye movement (REM) sleep deprivation. When the animals enter the REM sleep, they fell into the water due to muscle relaxation and consequently woke up [33].

In this study, the MMPM was designed to reduce individual stress, which included a recovery time window and provide social communication amongst animals without hindering free movement [22]. The tanks were filled with water to a level one cm below the surface of the platforms or the grid. The animal adaptation was done by placing them in the apparatus and subjected to sleep restriction protocol for 30 min daily for 5 days before initiating the CSD experiment. The CSD was inflicted for 21 days through which animals were placed in the tank for 18 h/day, from 04:00 pm to 10:00 am and the next day, rats were returned to their cages, where they were allowed to sleep a 6-h window (10:00 am to 04:00 pm). The MMPM tank was cleaned carefully, and filled with water at a temperature between 20 and 25 °C before 4:00 pm. The grid floor control group contained animals placed on the top of the round platforms, but the surface of the water tank was covered by a meshwork of stainless steel, which we called it as grid floor. The grid floor prevented rats to fall into the water and so, they could sleep. This induced stress without sleep deprivation and can be considered as the control for the CSD group [33]. The rats in the control group were routinely fed and gavaged with PBS for 21 days and kept in their cages in the same room.

### Dissection of the neck for removal of the SCG

**Fig. 1:**
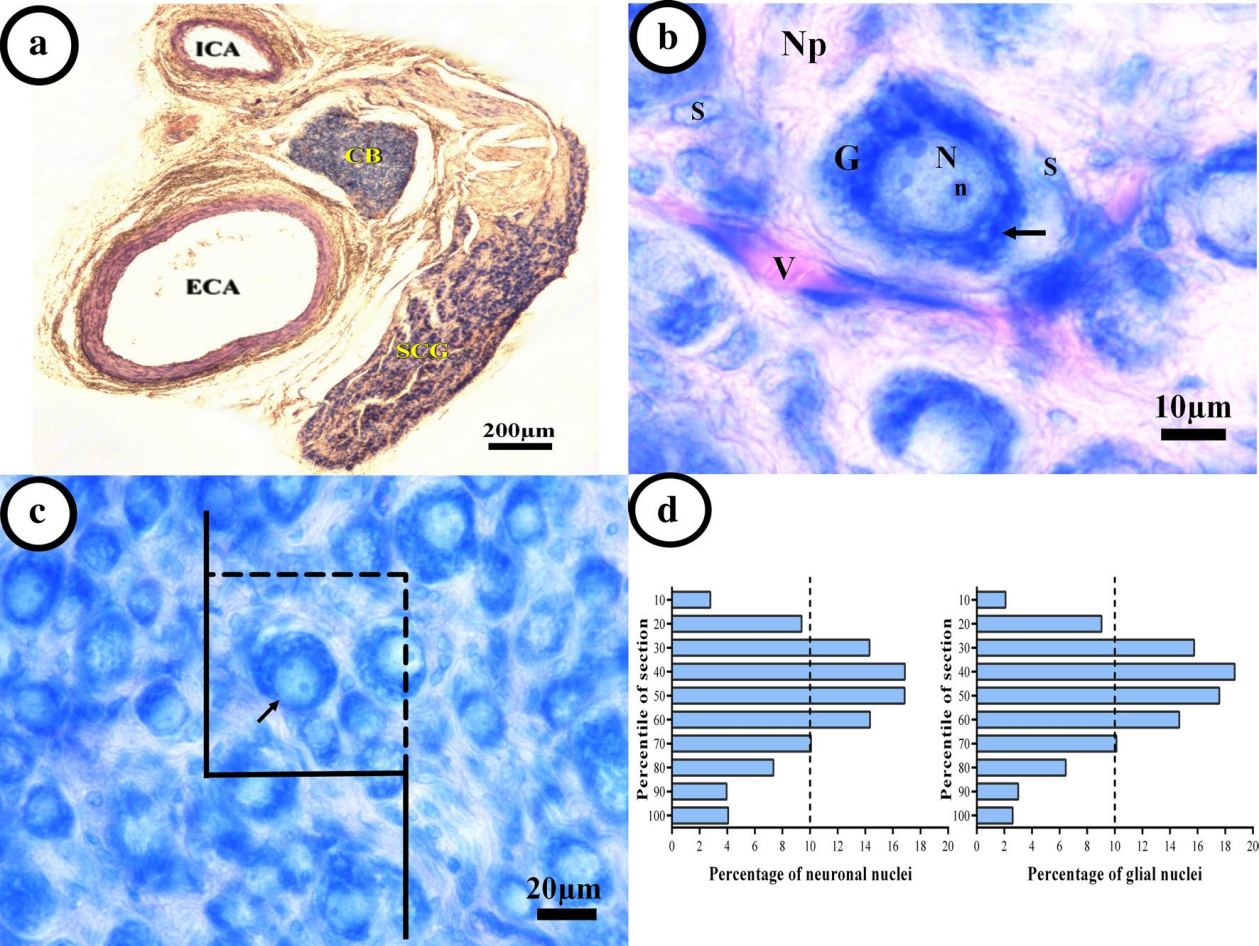
Stereological methods to evaluate the quantitative features of the superior cervical ganglion. **a** Position of the superior cervical ganglion (SCG) between external (ECA), internal (ICA) carotid arteries, and carotid body (CB). **b** Representative photograph from Giemsa-stained SCG shows the large ganglionic cell bodies (G), with nucleus (N) and nucleolus (n), Nissl bodies (arrow) in the neuronal cytoplasm. Sheets from small, elongated, or oval nuclei satellite cells (S) enclose each neuronal cell body and a vessel (V). Neuropil (Np) is a complex of neuronal process and connective tissue between the cells. **c** An optical disector snapshot. Neurons in which their nuclei came into focus during the scanning of the disector’s height without touching the left and bottom borders of the frame were counted (arrow). **d** The Z-axis distribution of the neuronal or glial nuclei was plotted to define the height of the disector. There are 10 columns each representing the percent of the counted nuclei in 10% of the section thickness from the top to bottom sections

After induction of deep anesthesia, all animals were sacrificed by the euthanasia method based on Guideline for the Euthanasia of Animals (by inhalation of chloroform) [34]. A vertical neck incision was made and the skin retracted. The superficial cervical fascia was removed, and then the salivary glands, external jugular veins, and the superficial muscles of the ventral neck were dissected. The cranial portions of the sternocleidomastoid and omohyoid muscles were transected and the common carotid artery, the internal jugular vein, and the vagus nerve were exposed. The common carotid artery was bluntly dissected up to the level of its bifurcation into the external and internal carotid arteries. The SCG is located behind the carotid bifurcation and freed of fat and connective tissues (Fig. 1a). Finally, the SCG was detached from the sympathetic chain and collected [35].

### Tissue preparation

After removing the bilateral SCG and fixation in buffer formalin, the right side SCG was paraffin-embedded, serially sectioned, and stained using Giemsa to estimate the number of neurons and glial cells as well as the volume of the SCG and its spatial distribution of neurons. Also, the paraffin-embedded left SCG was processed and sectioned for TUNEL assay. The stained sections were examined under a microscope by a researcher who was blinded to the experimental condition. It should be noted that the obtained ganglion volume might not represent the true absolute values and may include systematic errors due to shrinkage. However, shrinkage can be assumed to have an equal effect on the ganglia in all groups, and therefore, the obtained data could be used for comparing the six groups.

### The stereological methods

#### Estimation of the volume

The ganglions were longitudinally placed in paraffin molds, and serially cut into 25-µm thick sections and stained with the modified Giemsa method [36]. The volume of the right SCG was estimated using point counting based on the Cavalieri’s principle, using an Eclipse microscope (Nikon, E200, Japan) linked to a camera (Sony, Japan). Systematic random sampling was applied to samples with fixed intervals determined in advance (8–12 sections in each ganglion). The image of each section was assessed at a final magnification of 110× using the stereological software (StereoLite, SUMS, Shiraz, Iran). The volume of each ganglion (V_ganglion_) was estimated, using the following formula:

$${\text{V}}_{{{\text{ganglion}}\,}} = \,\sum {\text{A}}\, \times \,{\text{d}}$$

Where “∑A” is the sum area of sections and “d” is the interval between sampled sections [37].

### Estimation of the neuron and glial cell number

The number of neuronal and glial cells was counted in 8–12 sections of each ganglion using the optical disector method. Estimation of the number of satellite cells was obtained by counting all small dark nuclei with unclear cytoplasm while the ganglionic neurons appeared multipolar with large perikaryon, euchromatin nuclei, and prominent nucleoli. Individual ganglionic cells are enclosed by a capsule of satellite cells (Fig. 1b) [38].

A computer with the stereological software (StereoLite, SUMS, Shiraz, Iran) was linked to a light microscope (Nikon E200, Nikon, Japan) with an oil immersion lens (40×, numerical aperture: 1.3) which was utilized to estimate the total number of the neurons and glial cells. According to the “optical disector” technique, the microscopic fields were scanned and sampled by moving the microscope stage with equal distance in X and Y directions to ensure systematic uniform random sampling. The movement of the microscope stage in Z-axis was measured using a microcator (MT12, Heidenhain, Traunreut, Germany) installed on the stage [39]. The unbiased counting frames (Fig. 1c) with the area (“*a/f*”) of 3492 µm^2^ has been used for the neurons and glial cells of the SCG. To obtain suitable guard area and the height of the disector (h), Z-axis distribution from the nuclei was plotted. Briefly, the counted nuclei were grouped in 10 columns through the height of the tissue (calculated by the microcator) from the top (0%) downward (100%). The Z-axis distribution of the nuclei is presented in Fig. 1d. The upper 10% and lower 20% of the neuronal nuclei and the upper 20% and lower 10% of the glial cell nuclei were ignored based on the histogram, which were regarded as the guard zones. The mean focus distance to un-focus in all counted fields, based on which height is obtained in each column. Therefore, cell counting was carried out at the remaining 81% and 78% of the neuronal and glial cell nuclei, respectively within (h) and corrected for overestimation [33]. Any neuronal nuclei entering the focus within the sampling box (h multiplied by *a/f*) was selected if it was located totally or even partly inside the counting frame and also did not touch the unacceptable lines (left and bottom borders of the frame) (Fig. 1c). The total number of the neuron or glial cell was estimated by means of multiplying the numerical density (Nv) and V (SCG):

$${\text{Nv}}\left( {{\text{cells}} / {\text{unit volume }}} \right) = \left[ {\frac{{\sum {\text{Q}}^{ - } }}{{\sum {\text{P}} \times \left( {{\raise0.7ex\hbox{$a$} \!\mathord{\left/ {\vphantom {a f}}\right.\kern-0pt} \!\lower0.7ex\hbox{$f$}}} \right) \times {\text{h}}}} \times \frac{t}{\text{BA}}} \right]$$

Where “ΣQ^−^” was the total number of the nuclei coming into focus throughout scanning the height of the disector (Fig. 1c); “ΣP”, the total number of counting frames in all counted fields; “h”, the height of the disector; “*a/f*”, the frame area; “*t*”, the mean section thickness calculated in every sampled field, using the microcator (average 23 µm); and “BA”, the block advance of the microtome set at 25 µm [39]. The coefficient of error (CE) for the volume and number estimation was calculated based on Gunderson et al. [40].

### TUNEL assay

To evaluate cell death, the SCG sections were subjected to TUNEL assay using the in situ cell death detection kit, POD (Roche Diagnostics, Indianapolis, USA) according to the manufacturer’s protocol. The deparaffinized tissue Sections (5-μm mounted on superfrost slides) were hydrated in a gradually decreasing graded series of ethanol. Endogenous peroxidase was blocked by treatment with methanol containing 3% H_2_O_2_ and slides rinsed with PBS, 10 min for DNA decondensation, and incubated in a 50 µg/ml Proteinase K (Roche Diagnostics, Indianapolis, USA) in 10 mM Tris- HCl solution, pH 7.4–8.0, 45 min at 37 °C in a humidity chamber. After washing the slides with PBS, sections were incubated with the TUNEL reaction mixture for 60 min at 37 °C in the dark. The incorporation of the labeled solution was stopped by briefly rinsing the sections in distilled water and PBS. Sections were analyzed in a drop of PBS under a fluorescence microscope (Olympus, BX51, Japan) equipped with a digital camera (Olympus DP73, Japan), using excitation wavelength in the range of 450–500 nm and emission was detected in the range of 515–585 nm (green). Finally, converter-POD was added and samples were incubated at 37 °C for 30 minutes. Sections were incubated with 0.5 mg/ml diaminobenzidine (Sigma-Aldrich, USA) in 0.05 mol/L TBS (pH 7.6) with 0.02% H_2_O_2_ for 10 min, washed twice in distilled water, and lightly counterstained with fast green 0.1% before coverslipping. Each experiment included negative and positive controls. Negative control was performed using a labeled solution instead of TUNEL reaction mixture. The rat thymus with hydrocortisone-induced apoptosis thymocytes was used as a positive control [41]. For the quantitative analysis, the percentage of TUNEL-positive ganglion cells (neuron) was evaluated in 10 randomly selected fields by image J (Java. NIH, USA). The apoptotic index was determined by the equation below [42]:

$${\text{Apoptotic index }} = \frac{\text{apoptotic cell}}{\text{total cell}} \times 100\%$$

### Evaluation of spatial distribution of neurons

The spatial distribution of neurons in the superior cervical ganglion was evaluated with the Voronoi tessellation method, which was obtained by constructing Voronoi polygons. Each polygon encompasses the areas in which the cells are accumulated and close together. Thus, a polygon area indicates the spaces that a cell occupies. The area and the number of closest Voronoi polygons to each other were then obtained. To draw the Voronoi polygon diagram, tissue sections (with a 40× objective lens) of this ganglion were analyzed using the video-microscopy system. Each image was imported to the Image J software [32]. In the Voronoi tessellation, Voronoi polygons are of variable areas, some large and other small; the variability of polygon areas is easily assessed by their variance. The coefficient of variation, CV (standard deviation of the polygon areas/mean × 100) allows expressing the variability in a scale-independent manner. The CV that is a classification and not statistical comparison indicates the spatial distribution of neurons: CV of 33%–64% is associated with a random distribution, less than %33 has a regular distribution, and more than  %64 considered as a clustered distribution [43].

### Statistical analysis

Data are presented as Mean ± SEM for each experimental endpoint. The Kolmogorov–Smirnov test was done to determine the normality of data. For bodyweight, 2-way ANOVA and for apoptotic cells, one-way ANOVA assay was performed and followed by appropriate Tukey’s posthoc test for parametric (normality and equal variance passed) data. Kruskal–Wallis ANOVA based on ranks followed by Dunn’s posthoc test was used for nonparametric (normality and/or equal variance failed) data. Group differences in volumes, neuronal and glial cell numbers were tested using 2-tailed Mann–Whitney U Test to compare two pair groups. For the area of Voronoi polygons, a one-way ANOVA was conducted to assess the overall statistical significance of differences among the groups, and the Levene’s test in SPSS was used to determine the equality of variances. All *P* values < 0.05 were considered to be statistically significant. The statistical analyses were done using the GraphPad Prism 6 Demo (Version 6.07, © 1995-2015 GraphPad Software, Inc., USA) and SPSS (SPSS, Version 22, IBM Corporation, USA).

## Results

Animals were weighed on days 1, 7, 14, and 21 of the study. The result of bodyweight was analyzed by the following formula

$$\varvec{Body\,weight\% } = \left[ {\frac{{\left( {\varvec{weight \,of }21^{{\varvec{st}}} \varvec{ day}} \right) \times 100}}{{\varvec{weight \,of\, first\, day}}}} \right]\varvec{ } - 100$$

There was no significant difference regarding the bodyweight of rats across all groups at the beginning of the experiment. Bodyweight at day 21 increased by 18 and 19%, in the control and curcumin-treated group, respectively in comparison with the first day while 2.66% mean weight loss was observed in the sleep deprivation groups. Treatment with curcumin in the CSD group could not significantly prevent weight loss (data was not shown).

### The histomorphological evaluation

**Fig. 2:**
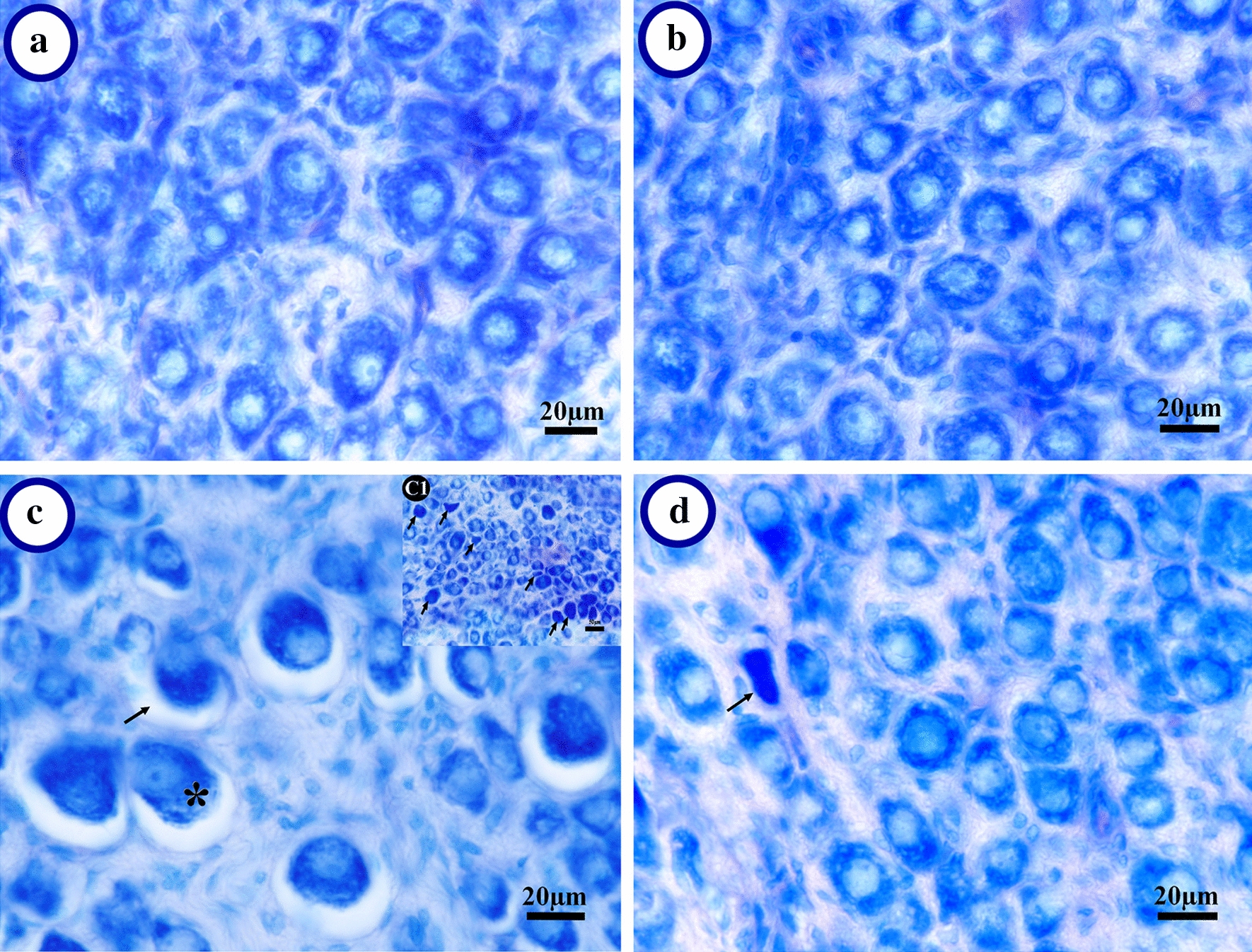
Representative photograph of the superior cervical ganglion (SCG). **a** Normal appearance in the cage-control SCG. **b** Normal appearance of ganglionic cells in curcumin-treated animals with more cells. **c** The chronic sleep deprivation (CSD) SCG: A less population of the ganglionic cell as well as the hypertrophied cells (*), increase of perineuronal space (**→**) could be seen. Also pyknotic cells (**→**) can be found in the CSD animals (**c**_**1**_). **d** The SCG in the CSD animals that received curcumin appeared to have remained similar to normal histology. Arrow indicates pyknotic cells

Histomorphology of the SCG is presented in Fig. 2. There was no evidence of adverse alterations in the size and density of neuron or glial cells, and perineuronal space on microscopic examination in the control group (Fig. 2a). Also, there were no significant changes in the histological appearance of the control group compared to the grid floor and grid floor + curcumin groups (data not shown). The normal rats that received curcumin, retained its normal tissue appearance, although it seems that neurons have relatively more density (Fig. 2b). The SCG in the CSD animals showed a lower cell population, neuronal hypertrophy, and expanded perineuronal space and more pyknotic cells in comparison with the control group (Fig. 2c). At the same time, with curcumin consumption during CSD, the SCG tissue preserved its appearance similar to the control group, although a small number of pyknotic cells are still observed (Fig. 2d).

### Volume of the SCG

**Fig. 3:**
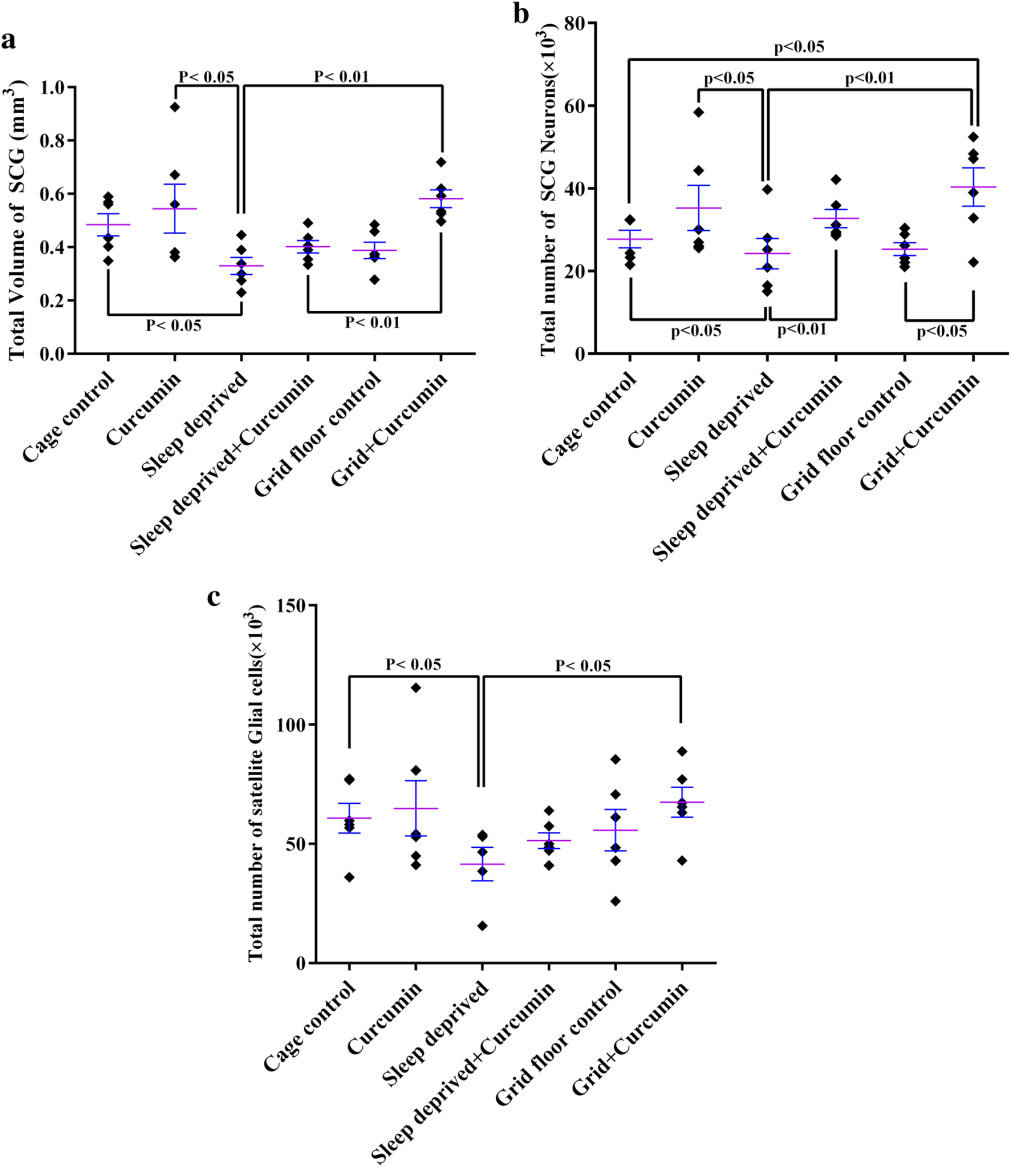
Dot plots showing stereological findings in the superior cervical ganglion (SCG) in different groups. **a** The total volume of SCG in different groups. The chronic sleep deprivation (CSD) significantly reduced the SCG volume and treatment with curcumin did not significantly improve this change. **b** The total number of neurons of SCG. The CSD non-significantly decreased this parameter and treatment with curcumin significantly improved this change. **c** The total number of satellite glial cells of SCG was significantly decreased in the CSD group in comparison with the control group and treatment with curcumin, although improved this change, however, it was not significant. The lines over each dot plot represent mean ± SEM (standard error of the mean)

The volume data are summarized in Fig. 3a. The estimation of the total volume of SCG did not show significant differences between the grid floor and control groups. This parameter in the chronic sleep deprivation (CSD) group was reduced to 31.93%, 39.44%, and 43.34% of the control, curcumin, and grid floor + curcumin groups, respectively (*P *< 0.05, Fig. 3a). This indicates that CSD can cause atrophy or cell loss in SCG. However, treatment of the CSD animals with curcumin have almost prevented these change in the SCG compared to the CSD group. All groups that had received curcumin showed a non-significant increase in the total volume of the SCG in comparison to their control groups (Fig. 3a), which might indirectly show the positive effect of curcumin on this parameter. The range of CE of the estimated volume using Cavalieri’s method was 4–5% for the six groups.

### Number of neurons

Figure 3b presents the total number of neurons in the SCG. The total neuronal number did not show significant differences between the grid floor and cage-control groups. The CSD led to a significant cell loss in the SCG compared to the control group (*P *= 0.04) and this parameter in this group was the lowest in comparison with other groups (*P *= 0 03 vs curcumin, *P *= 0 007 vs grid floor + curcumin, *P *= 0 007 vs CSD + curcumin). Treatment of CSD with curcumin prevented cell loss and retained this parameter to almost its normal level. The increase of neuronal numbers in the curcumin group when compared with the control group was not significant. Other groups that received curcumin showed more neurons in the SCG when compared with their control groups. This might be an indication of the curcumin protective effect. The range of CE of the estimated neuron number was 4–5% for the six groups.

### Number of satellite glial cells

The stereological estimate of the satellite glial cell (SGC) number in the SCG is presented in Fig. 3c. The total SGCs number did not show significant differences between the grid floor and cage-control groups. The total number of SGCs in the SCG had decreased by 31.74% in the CSD group as compared to the cage-control group (*P *< 0.05) and 38.5% vs grid floor + curcumin (*P *= 0.03). In the CSD animals that received curcumin, the number of glial cells showed similar to the control group. All groups that received curcumin, did not show a significant effect of this agent of the number of the SGC when compared with their control groups. Counting of these cells did not show significant differences between the other remaining groups. The range of CE of the estimated glial number was 4–5% for all groups.

### Percentage of apoptotic cells

**Fig. 4:**
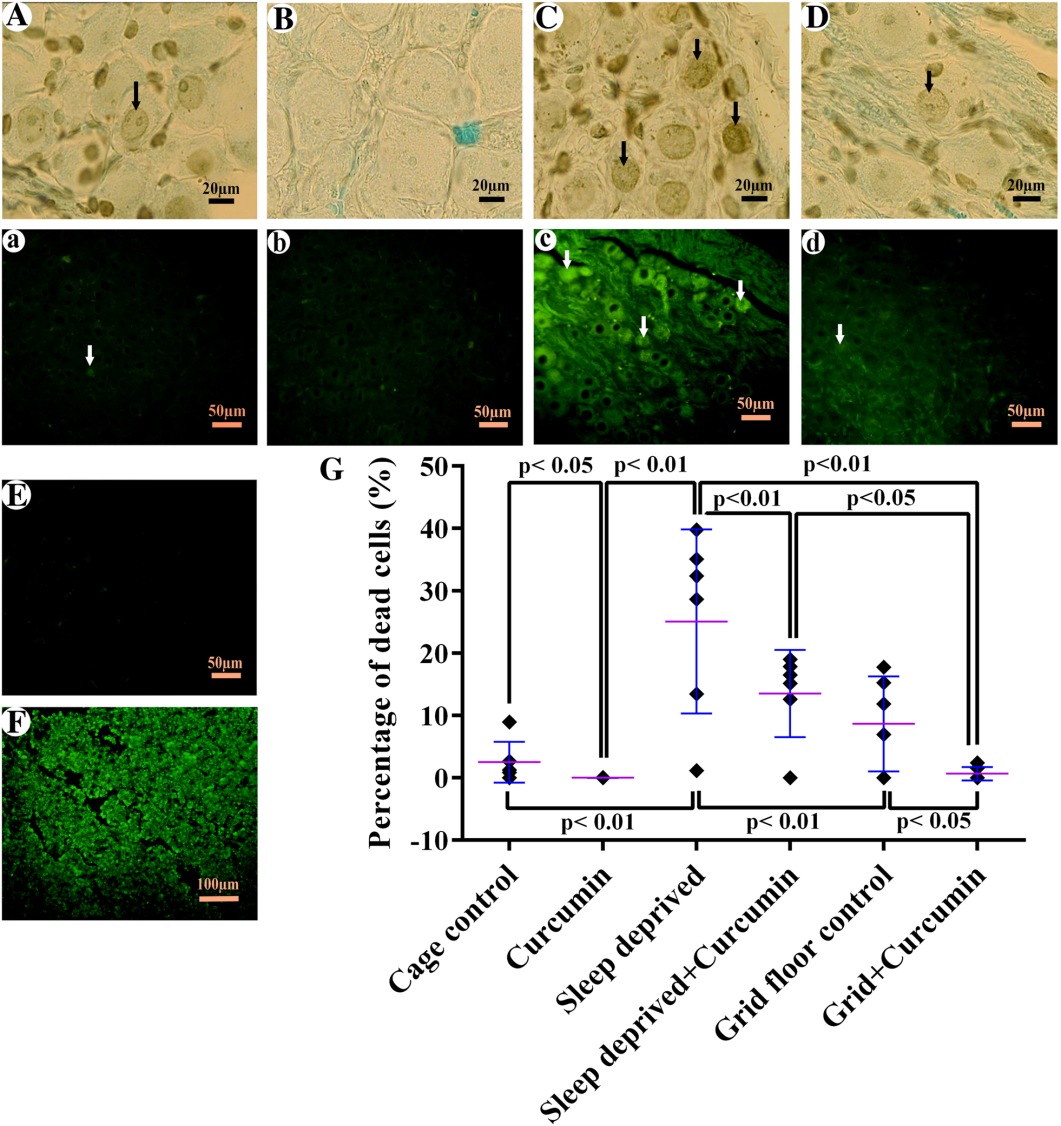
TUNEL assay of the superior cervical ganglion. **A**–**D** Diaminobenzidine detection by light microscope. **a–d** TUNEL assay with fluorescence microscope by excitation wavelength in the range of 450–500 nm; Arrow indicated apoptotic cells. **A&a** A normal appearance and low apoptotic cell can be seen in the cage-control. **B&b** In the curcumin-treated animals, the apoptotic cell was not observed. **C&c** An apparent increase in the number of apoptotic cells was observed within chronic sleep deprivation (CSD) animals compared with the control groups. **D&d** The number of apoptotic cells in the CSD animals receiving curcumin appeared to be similar to the control group. **E** The negative control, the TdT enzyme was removed from the TdT reaction buffer that did not display TUNEL-positive cells. **F** Positive control, the rat thymus with hydrocortisone-induced apoptosis thymocytes used as the positive control. A large number of apoptotic cells can be seen in this tissue. **G** The mean ± SEM of apoptotic cell percentage in the different groups

The result of the One Way ANOVA assay followed by an appropriate posthoc test (Tukey’s) showed a significant difference between treatment groups (*F*_5, 28_ = 19.68, *P *< 0.0001). The TUNEL-positive ganglionic cell statistically was similar between grid floor and cage-control groups, which, in association with stereological findings, indicates that stress of water tank on the rats, did not have a negative effect on this tissue. The percentage of TUNEL-positive ganglionic cells in the CSD group was significantly higher than the cage-control (90.03%, *P *< 0.0001). Treatment of CSD with curcumin reduced the number of dead cells (58.37%, *P *= 0.0008, Fig. 4g), and apoptotic cell number decreased in a way that was similar to the control group level that can be an indication for anti-apoptotic effect of curcumin. Also significant difference was observed between CSD group and grid floor control (65.5%, *P *< 0.0001), grid floor + curcumin (97.34%, *P *< 0.0001) and curcumin (95.35%, *P *< 0.0001). There was also a significant difference between curcumin and CSD + curcumin (91.38%, P = 0.017). Mann–Whitney assay showed the number of TUNEL-positive cells decreased significantly in healthy rats that had received curcumin (53.34%, *P *= 0.015). There is a significant difference between grid floor + curcumin and its control (92.29%, *P *= 0.03). Detection by fluorescence microscope (Fig. 4a–d) also reached similar results. The negative control sections did not display TUNEL-positive cells (Fig. 4e). The apoptotic cells in the positive control were observed, which proved our protocol (Fig. 4f). The mean ± SEM of the ganglionic apoptotic cell percentages has been shown with TUNEL in the rat SCG in Fig. 4g.

### Spatial arrangement of ganglionic neurons (Voronoi tessellation)

**Fig. 5:**
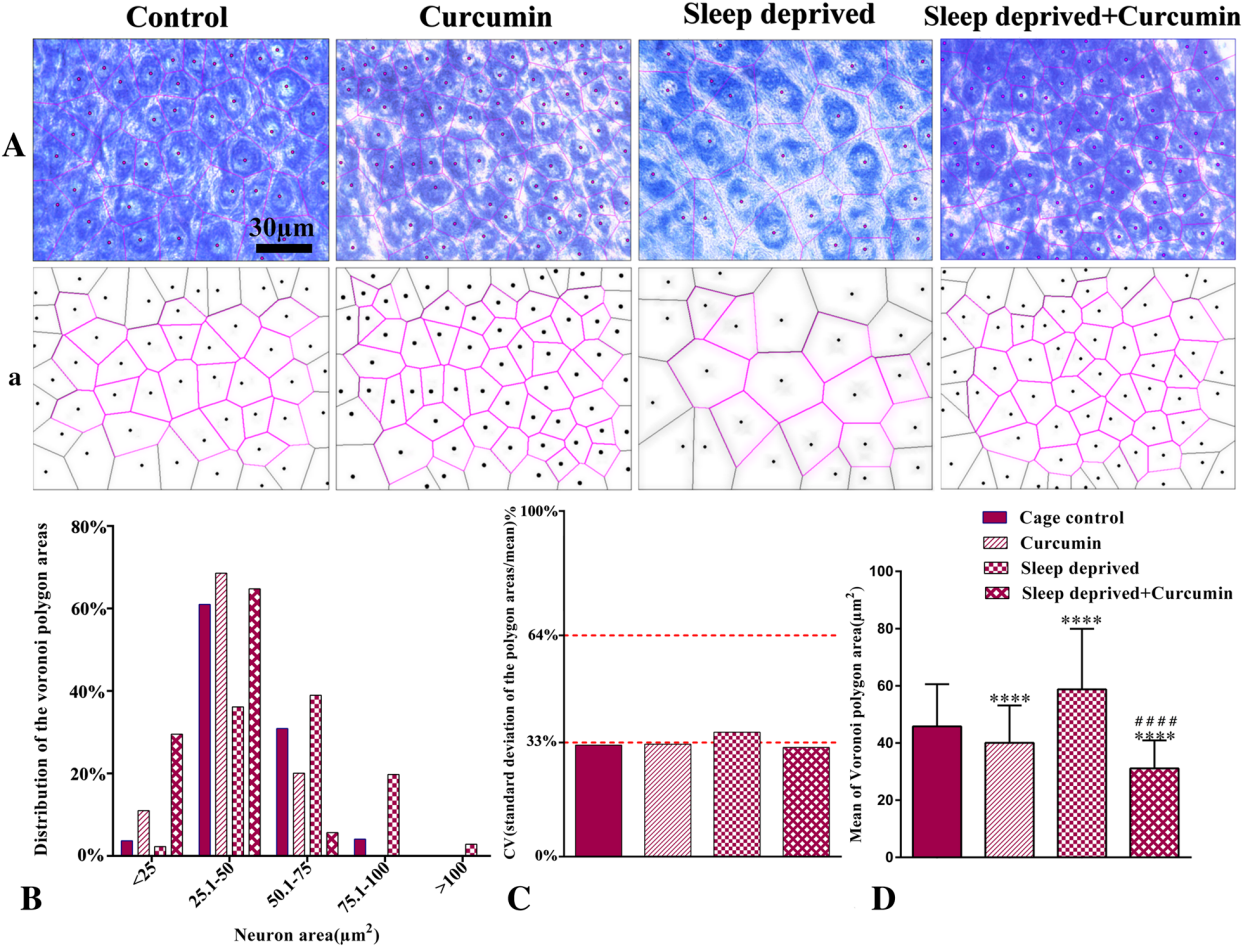
Representative photograph (**A**) and schematic (**a**) Voronoi tessellation of the superior cervical ganglion neurons in different groups. **b** The distribution of the Voronoi polygon areas. **c** The CV of the spatial distribution of neurons in different groups: CV (coefficient of variation) of 33%–64% are with a random distribution (sleep-deprived group) and less than %33 has a regular pattern (other groups). **d** The mean of polygon areas for each neuron (µm^2^) in different groups. **** *P *< 0.0001 vs. the control;#### P < 0.0001 vs. the sleep-deprived group

Figure 5 shows the Voronoi polygon areas calculated for the ganglionic neurons in different groups. Based on polygon area distribution, about 64% of the area of the polygons in the control group are placed in the range of 1–50 μm^2^, while 38% of neurons in the CSD group are in the same range. In fact, in the control group, the distribution polygon area was shifted to the left; however, this distribution in the CSD group was shifted to the right (Fig. 5b). Polygon area distribution in both healthy and sleep-deprived animals that received curcumin was about 79% and 94%, respectively in the same range. In these groups, the distribution polygon area was shifted to the left. The mean coefficient of variation (CV) of polygon areas was 32.27%, 32.53%, 35.99%, and 31.57% in the control, curcumin, CSD, and CSD + curcumin groups, respectively. These data showed that the distribution of neurons in control, curcumin, and CSD + curcumin groups were regular (CV < 33%) whereas the CSD group had a random distribution of neurons (CV > 33%, Fig. 5c). Although the report of CV is a classification, based on Levene’s test, there was a significant difference between the CV of all groups (*F*_3, 1016_ = 49.298, *P *< 0.0001). It means that Levene’s test showed that the variances for polygon areas were not equal. The mean polygon area of neurons significantly increased in the CSD group in comparison with the control group, however, treatment with curcumin significantly decreased this parameter when compared to the control group (*P *< 0.0001, Fig. 5d).

## Discussion

The present investigation was an attempt to examine the consequences of sleep deprivation with or without curcumin treatment on quantitative anatomical changes of the superior cervical ganglion in a rat model.

The first part of this study showed that CSD can lead to significant weight loss. The results of McHill and Wright [44] also showed both total sleep deprivation and REM sleep deprivation induce weight loss, exhibiting a state of increased energy expenditure. This suggests a change in normal metabolic function in sleep-deprived rats.

In the second part of our study, light microscopy analyses showed that the various morphological changes occurred during CSD. In the cage-control SCGs, Giemsa staining revealed that they contained ovoid to polygonal neuronal cell bodies with Nissl body in the cytoplasm without obvious perineuronal space, enclosed with a sheet form from small satellite glial cells (Fig. 2a) while in the chronic sleep deprivation (CSD) animals, a less population of ganglionic cells as well as some pyknotic and hypertrophied cells and expanded perineuronal space were found (Fig. 2c). In earlier studies on sensory ganglia, the perineuronal spaces in some of the neurons that were similar to the CSD group in the present study were suggested to be due to either shrinkage or apoptosis of neurons [45].

The third part of our results demonstrated that chronic REM sleep deprivation causes a significant reduction in the total volume of the SCG as well as a loss of ganglionic neurons and satellite glial cells. Reductions in volume due to sleep deprivation were previously found in the brain [46], prefrontal cortex [47], hippocampus [30], and thalamus [23]. Some explanations were proposed for SCG volume reduction observed in the SCG of CSD group including neuronal death, neuronal shrinkage, lower dendritic arborization, or decreases in glial cell numbers and even reductions in neurogenesis [48]. In the present study, the decrease in the number of neurons (12.63%) and glial (26.45%, Fig. 3) along with an increase in apoptotic cells in the CSD group (Fig. 4) might be the reason for the reduction in SCG volume. Volume reduction in this structure could be also attributed to the decrease in neurogenesis or neuronal dendrite arborization that should be clarified in future studies, although there are contradictory reports regarding the neurogenesis in SCG. On one hand, Melo et al. reported an age-related increase in the SCG volume and the total number of SCG neurons [49], and Ladd et al. along with the increasing SCG volume, showed the presence of new-born neurons in old animals by using BrdU technique [50]. On the other hand, Walters et al. recently showed that there is no evidence of active neurogenesis in sympathetic ganglia [51].

In this study, the number of ganglionic cells in the CSD decreased in comparison to the control group, which is almost in line with the previous studies that showed a decrease in the number of neurons in different parts of the brain, such as the hippocampus, dentate gyrus [52], and dorsal respiratory nuclei in the brainstem following the CSD [22].

Herculano-Houzel [53] stated that a similar task is performed better in the same species with more neurons. Fang et al. [54] found that animals with more cortical neurons also had enhanced functional correlations and more distinct neuronal ensembles in the primary visual cortex. These results suggest that neuronal numbers may be linked to functional modularity and cognitive differences.

Our findings, that CSD decreased glial cell number in the SCG, is in line with the study of Roman et al. [8] who showed sleep deprivation reduced astroglia production and possibly a limitation of glial cell function, such as the release of growth factors. There is plenty of data on the physiological properties of SGCs. Common theories suggest that these cells have a notable role in controlling the microenvironment of the sympathetic ganglia, which are very similar to those of astrocytes. Hanani [38] indicated that not only the maintenance of the environment but the modulation of neuronal activity by bidirectional transmitter interactions are part of the functions of SGCs. SGCs almost completely envelop the neuron and can regulate the diffusion of molecules across the cell membrane. It was previously shown that SGCs can regulate the extracellular matrix of individual neurons [55]. These findings suggest that a decrease in the number of SGCs following sleep deprivation might have a role in peripheral and central nervous system diseases, although this should be clarified in the specific future studies.

The fourth part was conducted to answer this question, whether this decrease in the structure volume and cell number could be due to the increase in cell death?

Our TUNEL results indicated that cell death had increased due to CSD (Fig. 4), which is in line with the findings of previous studies. For instance, Biswas et al. [56] showed that apoptosis induced by REM-sleep deprivation occurred in the rat brain. They proposed that the activation of the mitochondrial intrinsic pathway, reduction in cytoskeletal proteins, alterations in neuronal cytomorphology, and the levels of pro-apoptotic and anti-apoptotic proteins in the rat brain are involved in this apoptosis. As shown in Fig. 2c and d, rats exposed to CSD had hypertrophic and pyknotic cells, although this change in size could be attributed to the expression of other cellular proteins, it might be a landmark for necrosis or apoptosis due to CSD, which is also in line with other studies [56, 57].

The fifth part has focused on the protective effects of curcumin by preventing anatomical changes that occur in the SCG of sleep-deprived animals. The SCG histological appearance in the CSD animals that received curcumin preserved similar to the control group (Fig. 2d). As seen in Figs. 3b and 4g, the larger number of neurons and a smaller number of TUNEL-positive cells in sleep-deprived rats receiving curcumin in comparison with the CSD groups, confirming curcumin in preventing cell death, which is in line with previous studies [30, 47]. The previous studies show that curcumin increases cell survival [58] and based on a recently published study, a lack of proliferation activity has been reported in the SCG [51]. Besides, by inhibiting pro-apoptotic factors such as Bcl-2-associated X protein [59] and activating silent information regulator 1 (SIRT1) [60] curcumin has antioxidant properties. Furthermore, curcumin, through SIRT1, has a key role in cellular metabolism and response to oxidative stress in mammals [61]. Also it has a neuroprotective effect in neurological diseases and brain injury [62]. Therefore, the possible mechanism of curcumin in preventing neuronal loss may be attributed to reducing apoptosis rather than inducing cell proliferation, and it may exert these effects through antioxidant activity.

The effects of curcumin in preventing apoptosis are in line with the previous studies, indicating the beneficial effect of curcumin in different CNS disorders. For example, Kumar et al. [63] and Bavarsad et al. [64] have described curcumin as a safe drug that acts as both neuroprotective and anti-apoptotic agent on age-related impaired cognition and memory and ischemia–reperfusion injury in the nervous system, respectively. Fan et al. [65] indicated that curcumin had a protective effect against IL-1β-induced neuronal apoptosis in chronically stressed rats, which might be related to its anti-apoptotic effect. Protective effects of curcumin on CSD-induced changes in some other parts of the nervous system, such as the hippocampus [30], prefrontal cortex [47] and dorsal respiratory nuclei [22] were also investigated; however, as far as we know, this is the first report on the SCG histomorphological parameter changes due to CSD and beneficial effects of curcumin on these changes.

Finally, we used Voronoi tessellation in this study that is a well-known mathematical cellular structure, which can approve and complete stereological evaluations. The Voronoi polygons allow calculating the confidence interval of an average numerical density that enables statistical comparison. Tessellation also provides spatial distribution information. Voronoi tessellation has been extensively studied but has been recently used to analysis of histological maps [43].

For the first time, we assessed the spatial arrangement of ganglionic neurons in the SCG after CSD and curcumin therapy. We observed that the arrangement of these neurons was regular in control groups and CSD changed this distribution into a random pattern. However, CSD animals that received curcumin, showed regular distribution (Fig. 5c). There are no studies regarding the spatial arrangement of these neurons in SCG to improve our knowledge or compare. However, Sarkala et al. [66] showed that the spatial arrangement of CA1 hippocampal pyramidal neurons was regular in the healthy rats and it changed to random pattern after the brain ischemia. Furthermore, the mean area of Voronoi polygons in the CSD group showed larger neurons than control and curcumin-treated CSD groups (Fig. 5d) in the SCG. To the best of our knowledge, there was no other study that has evaluated the size of neurons in the sleep deprivation. However, McEwen [67] indicated that chronic stress such as sleep deprivation could lead to both hypertrophy (in the amygdala) and atrophy (in the hippocampus and prefrontal cortex) of neurons. Physiologic hypertrophy is usually adaptive and improves function. Pathologic hypertrophy may be adaptive in some situations but often results in changes in gene expression that can exacerbate organ dysfunction [68]. Cell swelling or hypertrophy may also be a sign of cell death [69]. Curcumin treatment has been shown to cause cell proliferation in previous studies, which significantly increased the number of Voronoi polygons in this study and reduced their mean area. The main effect of curcumin treatment is likely to be a dramatic reduction in cell death, which confirms the results of TUNEL.

In spite of the findings in the present study, it is still unclear whether such side effects of CSD on the nervous system and the beneficial effects of curcumin on this system are stable in the long term. To answer these questions, future studies are necessary. Furthermore, the effects of these ganglion structural changes on the function of circadian, cardiovascular, digestive systems should be considered in future studies.

## Conclusion

The present study indicated that CSD induced by the MMPM approach for 18 h/day over 21 days, results in body weight loss, and volume reduction, neuronal and glial cell loss and apoptosis in the SCG in a rat model. Oral consumption of curcumin could prevent neuronal loss and apoptosis in this ganglion, although its effect on changes in this structure volume and glial cell number is not significant. Finally, for the first time in the present study, we reported that the spatial arrangement of SCG neurons in the CSD animals changed into a random pattern, and using curcumin preserved its distribution similar to the normal structure in the regular one.

## Data Availability

All data generated and analyzed during this study are included in this published article.

## Abbreviations

CE: Coefficient of error
CSD: Chronic sleep deprivation
CV: Coefficient of variation
FDA: Food and Drug Administration
MMPM: Modified multiple platform method
SCG: Superior cervical ganglion
SGCs: Satellite glial cells
TUNEL: Terminal deoxynucleotidyl transferase-mediated dUTP nick end labeling
